# Supplementary material for: Extracellular Vesicles Derived from Young Neural Cultures Attenuate Astrocytic Reactivity In Vitro
Source: Int J Mol Sci. 2022 Jan 25;23(3):1371. doi: 10.3390/ijms23031371 (PMC8835866; doi:10.3390/ijms23031371)
Supplement: Supplementary file 1 [file ijms-23-01371-s001.zip › ijms-1521777-supplementary.pdf]

Extracellular vesicles derived from young neural cultures attenuate astrocytic reactivity *in vitro*

Daniel Almansa[1], Héctor Peinado[2], Raquel García-Rodríguez[1], Álvaro Casadomé-Perales[1], Carlos G. Dotti[1*], Francesc X. Guix[1*]

[1] Molecular Neuropathology Unit, Physiological and Pathological Processes Program, Centro de Biología Molecular Severo Ochoa, CSIC/UAM, Madrid, Spain.
[2] Microenvironment and Metastasis Group, Molecular Oncology Program, Spanish National Cancer Research Centre (CNIO), Madrid, Spain.
* Correspondence: **fguix@cbm.csic.es or fguixrafols@gmail.com** (Tel.: +34 911 96 4543) **cdotti@cbm.csic.es** (Tel.: +34 911 96 4519)

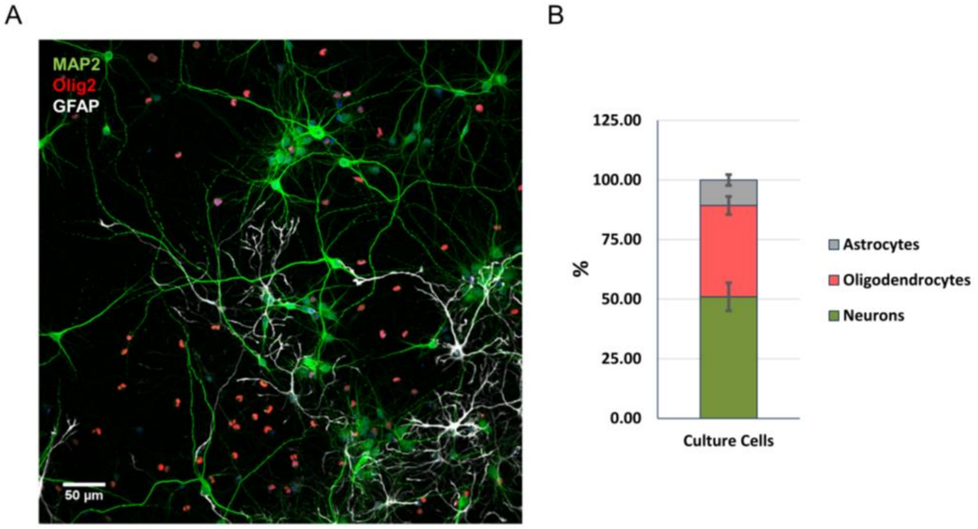

Supplementary Figure S1. (**A**) Representative confocal image of a 14 DIV rat primary culture from the cerebral cortex of E18 embryos, showing the presence of neurons, (MAP2, green), oligodendrocytes (Olig2, red) and astrocytes (GFAP, white). (**B**) Graph showing the relative amount of the three types of cells detected in panel A, quantified from 3 independent cultures. The graph shows the average relative amount (%) ± SEM.

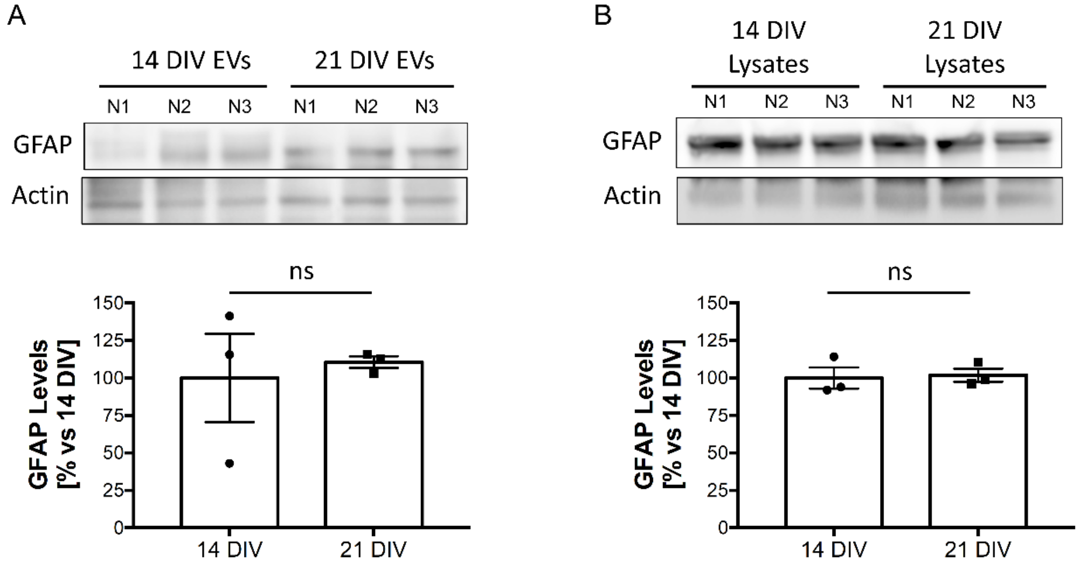

Supplementary Figure S2. (**A**) Western blot analysis of GFAP levels in extracellular vesicles (EVs) isolated from the media of 14 DIV and 21 DIV neural cultures (3 independent experiments indicated as N1, N2 and N3). The same amount of total protein (25 µg of EVs sample) was loaded onto the gel. Actin was used as a loading control. Below, the graph compares the relative (%) levels of GFAP between 14 DIV and 21 DIV EVs, quantified from the bands of the western blot shown in this panel. (**B**) Western blot analysis of GFAP levels in total lysates obtained from 14 DIV and 21 DIV neural cultures (3 independent experiments indicated as N1, N2 and N3). Actin was used as a loading control. Below, the graph compares the relative (%) levels of GFAP between 14 DIV and 21 DIV neural cultures, quantified from the bands of the western blot shown in this panel. All graphs show the mean protein levels relative to 14 DIV neural cultures ± SEM. Statistical significance was analyzed by two-tailed unpaired t test (ns = non-significant, N = 3).

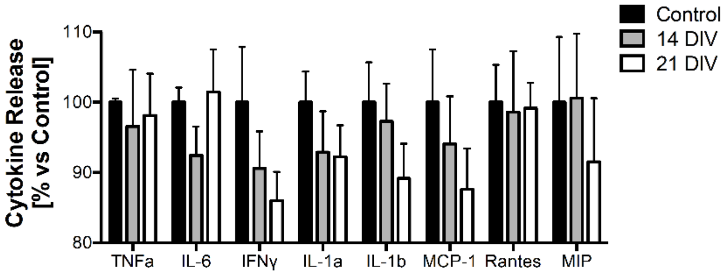

Supplementary Figure S3. Plot comparing the release of different cytokines to the media of 14 DIV neural cultures untreated (control) or treated for 24h with EVs isolated from the media of 14 DIV or 21 DIV neural cultures. Graph shows the mean levels relative to control ± SEM. Note: the y-axis starts at 80% in order to better appreciate the differences between conditions. None of the changes were statistically significant by two-way ANOVA analysis.

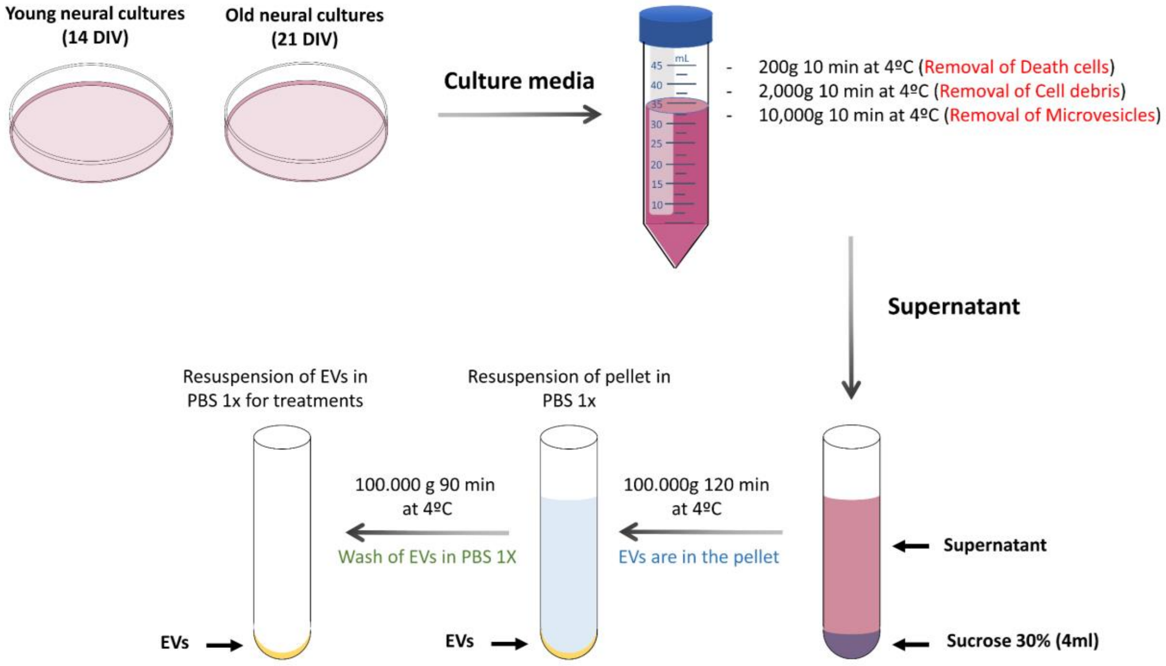

Supplementary Figure S4. Scheme showing the protocol for extracellular vesicles (EVs) isolation. The media of 14 and 21 DIV rat cortical neural culture (9,6 x 10^6 cells) was centrifuged at 200g for 10 min at 4 °C and consecutively at 2,000 g for another 10 min at 4 °C, to remove death cells and cell debris, respectively. A 30 min centrifugation at 10,000g and 4 °C was applied to the resulting supernatant (SN) to eliminate most of the microvesicles. Next, 34 ml of the SN was applied on the top of a 4 ml layer of a 30% sucrose solution in PBS, intended to better preserve the EVs structure (63), and centrifuged at 100,000g for 120 min and 4ºC. The 30% sucrose layer containing EVs was recovered and washed with ice-cold PBS (32 ml) and centrifuged for 90 min. Finally, the pellet was resuspended in 300 µL ice-cold PBS.
